# Product News

**DOI:** 10.1155/S1463924683000310

**Published:** 1983


Journal of Automatic Chemistry, Volume 5, Number 2 (April–June 1983), pages 114–120

### Product News

### Large-capacity electronic balances

Fisher Scientific have announced two large-capacity electronic top-loader balances: the LC-1000, with 1000 g capacity and ±0·01 g sensitivity, and the LC-5000, with 5000 g capacity and ±0·1 g sensitivity. Both balances feature extra-large LCD displays which are easy-to-read and generate little heat build-up that can interfere with stability. They automatically correct for such weighing variables as temperature changes and electrical fields and permit full-range taring. Readings take 3 s.

*Fisher's Bulletin No. 639 describes the balances and is available from Fisher Scientific Company, 711 Forbes Avenue, Pittsburgh, Pennsylvania 15219, USA. Tel.: 412 562 8546.*

Circle No. 3 on Reader Enquiry Card

### Haematology quality control

An extension to Technicon's H6000 automated haematology system was announced in February. The extension has been called the 'C' package—the whole system now becomes the H6000/C. Up to five different control materials and five calibrators can be monitored—bar-code identification numbers especially reserved for the materials are used to initiate the calibration cycle. Ten parameters per control and four per calibrator can be tracked, and 200 control cup results can be held in store (40 cups on each of the five different control materials)—this eliminates the need for manual collation and plotting of data and allows continual and automatic updating of the QC data files.

Should the same calibration material be aspirated consecutively (up to 20 times), the system averages the values obtained to give greater overall precision. Batch, cumulative and daily data summaries are available, as well as plot charts of SD from label value or from the mean. The average values for calibration constants are stored to monitor machine stability and provide early warning of potential system problems.

Most quality control 'add-ons' tend to be designed to augment existing instrument-checks and to enable the performance of both short- and long-term statistical analysis on control and patient samples, patient and laboratory normal limits and ranges. Technicon describe the H6000/C as going much further. For example with autocalibration, increased sample data storage, and full patient demographics linked to a positive sample identification system.

Cumulative means and plots can be generated for patient data. In addition, 'Bull' moving averages monitoring up to five selected parameters can be produced. The groups of result averages are stored to provide a data-base for the evaluation of trends in patient population means.

Normal range limits are programmable by the operator and can be separated into 'male' and 'female' if sex-dependent variation occurs. This facility allows the automatic flagging of high and low abnormals on the patient report form. In the same way, 'Alert' limits may be entered by the user, enabling instantaneous warning on the VDU screen and recall for further examination of grossly abnormal samples.

A feature especially highlighted is the H6000/C's ability to collate full patient demographics with associated haematological results. Demographic data (machine sequence number, name, comments, age, sex, patient location [up to 99 locations], clinician's name [up to 99 names]) are entered by the operator; the latter four by means of two-digit codes. All demographics are automatically printed on the patient report form to save time and avoid the risk of transcription errors. Automatic file search, recall and edit facilities are initiated by entering sequence number, sample bar code number or patient hospital number, and, if necessary, employing patient's location and clinician's name. Edited results are indicated by the presence of the letter 'E' shown against the altered data.

In order to facilitate the loading of sample trays a printed copy of sequence or bar code number plus patient names may be used—to improve security and speed data handling. Processed results, together with associated demographics (up to a total of 250 patients), may be stored on file prior to sample processing. At any time a status report may be generated in order to show the remaining file storage space, avoiding overflow and protecting the data already on file.

*More information on the H6000/C from Technicon Instruments Company Ltd, Evans House, Hamilton Close, Basingstoke, Hampshire RG21 27E, UK. Tel.: 0256 29181.*

Circle No. 4 on Reader Enquiry Card

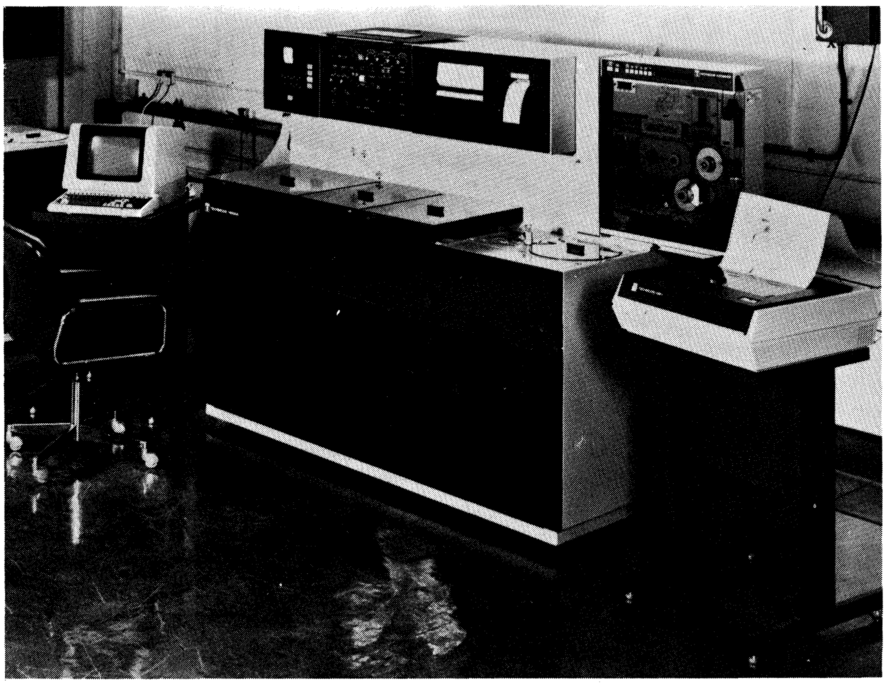

*The H6000/C. The package includes a programmable laboratory information system interface, this enables formatting flexibility, variable baud rate and choice of parity and control characters. A wide range of laboratory computers and printers can therefore be interfaced to the H6000/C. (Technicon Instruments Company Ltd, Basingstoke, UK.)*

### Detector for gas chromatography

Described as a revolutionary detector and launched in March, the Ion Trap Detector or ITD is both a universal and selective detector and is installable on any type of capillary gas chromatograph. The ITD enables the chromatographer to obtain positive identification of compounds in complex mixtures and to do so in many cases with only a single analysis. This contrasts with the usual procedure of performing several analyses employing specific GC detectors, such as FID (flame ionization), ECD (electron capture), or NPD (nitrogen phosphorus). The ITD delivers data in several easy-to-use forms familiar to the chromatographer, including chromatograms, selected mass chromatograms, mass spectra and quantitation reports. The detector operates in conjunction with an IBM personal computer; a simple one-letter command initiates any of its operations and the micro accepts all standard IBM software programs and peripherals.

Based on the 'ion trap' principle, the ITD is in some ways analogous to a mass spectrometer in its operating principle, but it differs greatly in its design and simplicity. It combines an ion source and mass filter in a single device without the need for ion-focusing lenses. It is suited to the analysis of compounds amenable to gas chromatography and within the mass range of 10 to 650 u. It scans at any of four sampling rates between 0·5 s and 4 s; in addition to a full scan mode the ITD can operate in a compound-selective or compound-class-selective mode by monitoring ions unique to the desired compound or class.

*More information from Finnigan MAT, 355 River Oaks Parkway, San Jose, California 95134, USA. Tel.: 408 946 4848.*

Circle No. 5 on Reader Enquiry Card

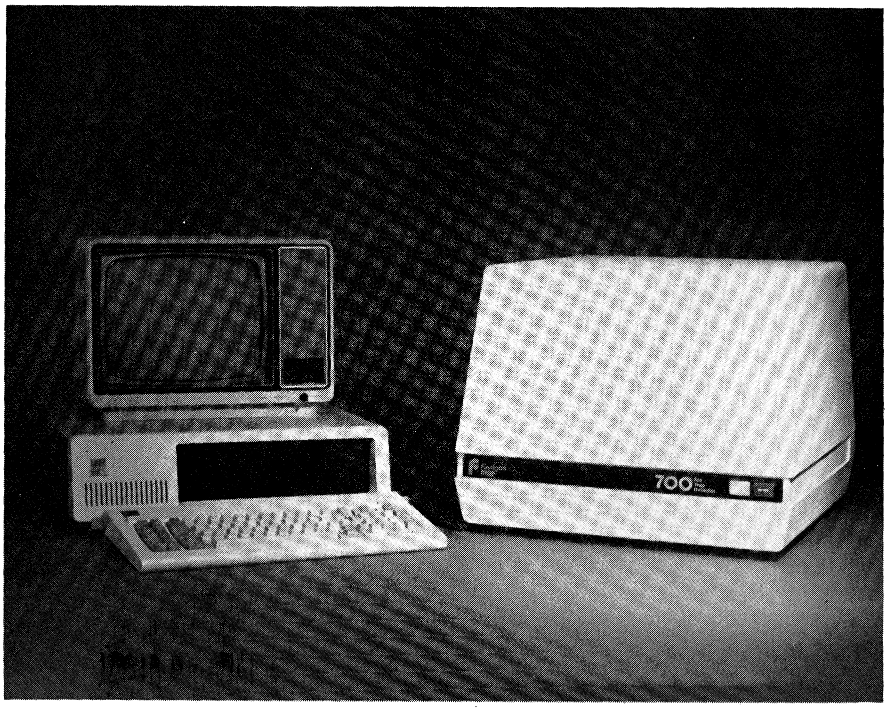

*Finnigan MAT's ITD, which was announced at the Pittsburgh Conference. Typical application areas are expected to be methods development to support routine GC analyses, industrial hygiene for positive identification of target compounds and unknown peaks, priority pollutant analyses for environmental applications, and clinical/biomedical monitoring.*

### High-speed anion analysis

Common anions can be analysed in 4 min with the Wescan anion high-speed column, which is now available in the UK from Techmation.

It is recommended for method development or routine analysis where separation speed or sample throughput are critical, and is complementary to the Wescan Model 260 series of single column ion analysers. Incorporating state-of-the-art microparticle HPLC column technology, the surface chemistry of this new anion/HS column matches that of the standard Wescan anion exchange column. So existing methods can be transferred directly to the new column without modification of the eluent composition.

*For an illustrated brochure and more information contact Techmation Ltd, 58 Edgware Way, Edgware, Middlesex HA8 8JP, UK. Tel.: 01 958 3111.*

Circle No. 6 on Reader Enquiry Card

### Refrigeration control with gas alarm

An instrumentation package for a refrigeration plant, which combines all the usual control functions with automatic safety monitoring of refrigerant gas levels, has been developed by Hall-Thermotank Ltd of Dartford, UK. The package was designed for a brewery and is thought to be the first of its kind. The unit is based on a standard Halltherm Redicon refrigeration plant control panel and incorporates a Crowcon Gaswarden 74GW gas detector—a modular unit with the capacity for up to eight separate channels. For this application the Gaswarden is equipped with seven monitoring channels, linked to seven sensors in the primary refrigeration plant. The Halltherm Redicon panel monitors, controls and logs refrigeration temperatures, pressures and so on, and the gas detectors sample the air at key points in the plant.

A two-speed flameproof fan in the plant's extractor ductwork, operates continuously at its lower speed to maintain a minimum of 15 air changes in the plant every hour. One of the gas sensors in this duct continuously samples the percentage of ammonia by volume in the extracted air: if this percentage reaches a pre-set level, the fan automatically is switched to its higher speed. If the gas level rises to another, higher, pre-set level then the plant is shut-down automatically and an alarm is sounded. The other gas-sensing heads are located at selected points in the plant where any significant gas leak would quickly be detected, and each control module is set to initiate immediately the alarm/shut-down sequence as appropriate to the gas level at that point.

In all instances, any operation of the gas alarm system, at either level, is logged and printed out as part of the permanent operating record provided by the package.

The instrumentation panel was built by Newlyme Controls Ltd, Newcastle-under-Lyme, UK.

*Further information from Crowcon Instruments Ltd, Temple Road, Cowley, Oxford OX4 2EL, UK. Tel.: 0865 776707.*

Circle No. 7 on Reader Enquiry Card

### Laboratory automation

The HP 3357 offers 33% more data-handling capability than the previous HP 3350 laboratory automation systems. It incorporates Hewlett-Packard's RTE-6/VM operating system so it allows the analytical laboratory manager to optimize productivity of people and equipment; in addition, the HP 3357 can schedule work, track laboratory supplies, maintain client accounts and generate activity summaries automatically. Turnkey data acquisition, chromatographic data reduction, control of automatic samplers and file management are some of the features of previous generations of HP systems. Recent software advancements incorporated into the HP 3357 broaden the capabilities of this HP 3350 series laboratory-automation system (LAS). Placing the software under control of a *session monitor* improves overall operating security and gives better control. This is accomplished by accountability through log-on allocation and variable security levels for RTE and file-manager commands.

*Expanded autocall and stream capability*: autocall and stream are no longer limited to LAB BASIC programs—any program compiled and stored on a disc may be autocalled or streamed.

Height calculation—in the past, by using peak 'AREA integration' as the calculation base, five standard chromatography calculations could be done: area-percent with and without named peaks, normalization, external-standard and internal-standard. These same calculations now can be performed using 'HEIGHT integration' as the calculation base, increasing the number of chromatographic-calculation procedures to 10.

*On-line quick reference*—to simplify user operation, a HELP file is available to obtain explanations of LAS error codes or assistance in using system commands.

*Wider sampling-rate bandwidth*—rate bandwidth has been increased from 60 HZ to 80 Hz, resulting in a 33% increase in throughput rate.

*16 Hz A/D sampling*: the rate at which the computer captures data from the A/D converter has been increased from 8 Hz to 16 Hz; this enhances the system's ability to perform analyses with peaks <0·5 s wide at half-height.

*DS/1000 IV compatibility*—this hardware/software product allows the HP 3357 to communicate with other HP 1000 and 3000 computers.

*Graphics/1000II*—a more powerful version of the HP 3356 Graphics 1000, the Graphics/1000II has three-dimensional figure manipulation and device independence. And in addition to providing operational support for Hewlett-Packard's new language systems, FORTRAN 77, MACRO/1000, and Graphics/1000II, the RTE-6/VM offers three new features that enhance programming productivity: Virtual memory for data; Shareable extended memory areas; and Extended code space.

*For a description of the system and its accessories contact the Enquiries Section, Hewlett-Packard Ltd, King Street Lane, Winnersh, Wokingham, Berkshire RG11 5AR, UK. Tel.: 0734 784774.*

**Circle No. 8 on Reader Enquiry Card**

### Catalyst analyser

The Micromeritics Instrument Corporation, who announced their 20th anniversary last year, have launched an automatic instrument that permits evaluation of catalyst surface activity at every stage of a catalyst life, from development and manufacturing to quality

**Circle No. 12 on Reader Enquiry Card**

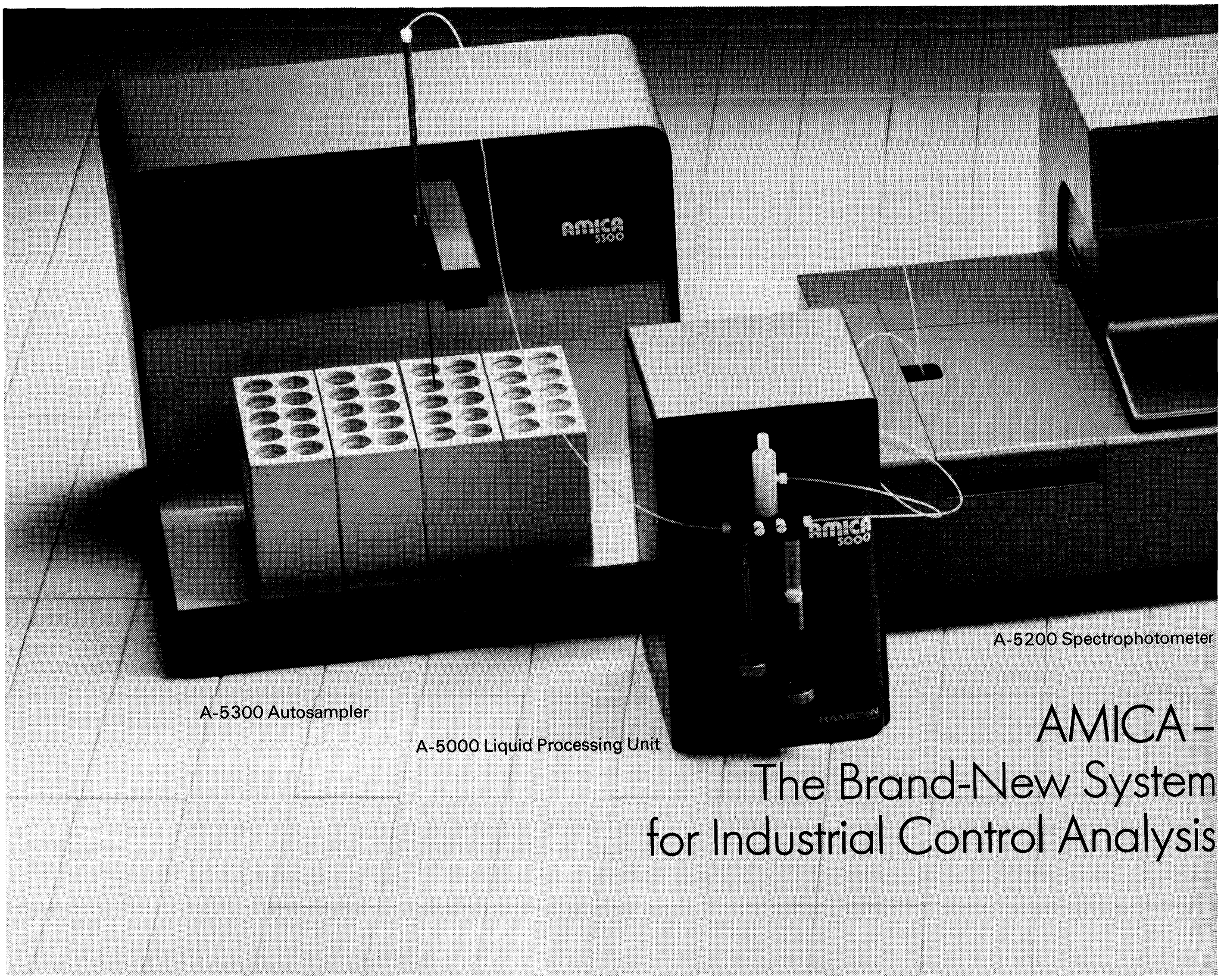

control and recovery. The ChemiSorb 2800 employs an improved chemical adsorption technique that allows multi-sample preparation and analysis.

Aimed at catalyst developers, manufacturers and users the unit provides information about the sites of heterogeneous catalysts, measuring the volume uptake of chemisorbate gas. This enables the calculation of both the reaction efficiency and expected life of a given catalyst. Automated sample preparation, instrument operation, and data acquisition and reduction substantially reduces the need for highly trained operators and increases the quantity and quality of analyses possible.

Operation is completely unattended: the operator loads the samples and is prompted by the instrument to enter the appropriate parameters for both sample preparation and analyses. Then the instrument automatically prepares and analyses up to five samples per loading. The computer records, reduces and reports all data.

*More information from the Micromeritics Instrument Corporation, 5680 Goshen Springs Road, Norcross, Georgia 30093, USA. Tel.: 404 448 8282*

Circle No. 9 on Reader Enquiry Card

### New journal

Covering all drug-related topics in pharmaceutical, biomedical and clinical analysis, Pergamon's *Journal of Pharmaceutical and Biomedical Analysis* was launched at the beginning of the year. The 'editors-in-chief' are Anthony F. Fell of Heriot-Watt University and James N. Miller of the University of Technology, Loughborough; they are supported by a large editorial board. Forthcoming papers include:

J. Goto *et al.* on 'Simultaneous determination of cortisol and cortisone in serum by HPLC and fluorescence detection'

T. C. O'Haver on 'The microcomputer revolution'

Y. Y. Z. Farid *et al.* on 'An assay for methotrexate and its metabolites in serum and urine by ion-pair chromatography'.

Annual subscriptions (the journal is quarterly) cost US$60 and £30.

*Specimen copies from Ingrid Petrauskas, Marketing Manager: Physical Sciences, Pergamon Press Ltd, Headington Hill Hall, Oxford OX3 0BW, UK.*

Circle No. 10 on Reader Enquiry Card

### Transit-time sonic flowmeters

Two bulletins, called SFM-2002 and SFM-2003, have been published by Mapco Inc. (Tulsa, USA) to explain quantitatively the performance and meter factors of transit-time sonic flowmeters. These flowmeters measure the difference in velocities of ultrasonic pulses travelling alternatively in the same and opposite direction as the flow of liquid in the pipe. It is the direct measurement of sound velocities to determine liquid velocities that make transit-time sonic meters very predictable. And this predictability has permitted reliable application of sonic meters to diameters of up to 120 in, which is beyond the limits of existing calibration facilities.

The application of sonic flowmeters in kit form to existing piping in the field is also made possible by this ability to predict meter factor from dimensions and liquid conditions. Sonic flowmeter kits are offered by Mapco for concrete, plastic and metal pipes.

*The bulletins are available from Mapco Inc., 11391 East Tecumseh Street, PO Box 21418, Tulsa, Oklahoma 74121–1418, USA. Tel.: 918 438 1010.*

Circle No. 11 on Reader Enquiry Card

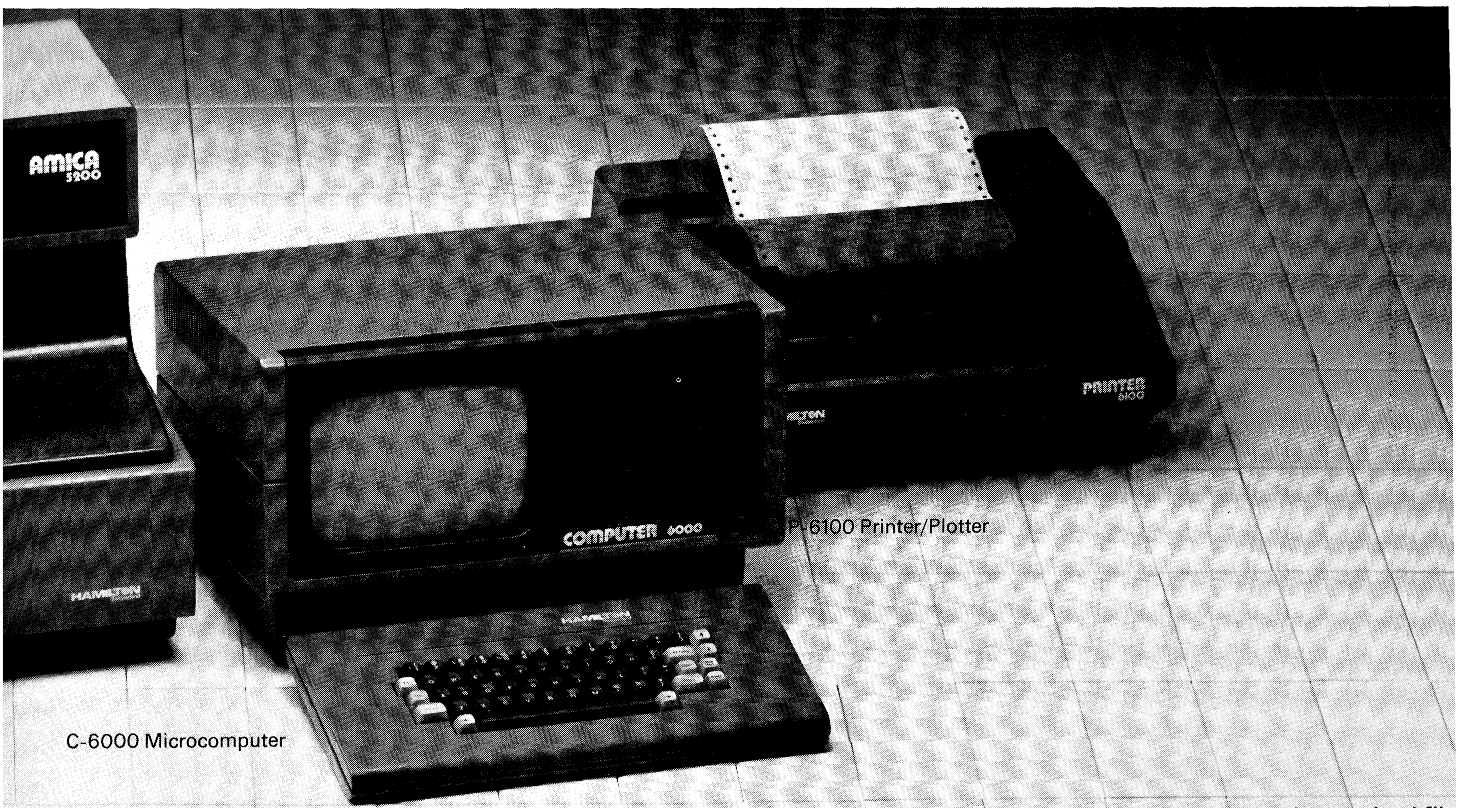

AMICA is an acronym meaning "Automated Modules for Industrial Control Analysis", an original concept for a new line of photometric and titrimetric analyzers.

### Protein/nitrogen analysers

The Nitrofoss semi-automatic Kjeldahl system was introduced to meet the need for analysis equipment resulting from requirements for tighter control on protein levels in the food and animal feed industries. The Nitrofoss range comprises two block digestors enabling maximum four and eight digests to be carried out simultaneously with an option switch for two or four respectively. These digestors incorporate radiant heaters giving a much better heating control than ordinary block digestors; the simmerstat control can operate up to a temperature of 625°C. Digestion can be carried out in the open laboratory because of an efficient self-contained fume extraction system. The distillation unit apparently takes all the danger out of diluting the digests, neutralization and steam distillation. All these procedures are carried out automatically. Final emptying after complete distillation is also automatic. This unit can also be used to determine cereal and milk protein by direct distillation or by Ronald's method. Additional equipment can be added to enable photometric determination of low nitrogen levels and determination of the chemical oxygen demand of water and sewage to be carried out.

*A full range of application notes covering the true Kjeldahl analysis, protein by direct distillation, nitrogen determination in fertilizers is available from Foss Electric (UK) Ltd, The Chantry, Bishopthorpe, York YO2 1QF, UK. Tel.: 0904 707944.*

**Circle No. 13 on Reader Enquiry Card**

### TLC applicator

Automatic and reproducible movement to the next application position is one of the features of the new PS 01 applicator, which has been developed by Desaga for use in thin layer chromatography. The movement is controlled by means of easily interchangeable templates which are available for 5, 6, 9, 10, 12 and 15 mm application grids. The application process is also automatic, being triggered as the dosing unit is lowered onto the TLC plate. From 20 to 10 000 μl of solution can be applied in point spots with an accuracy of < 1%. For larger application volumes, the solution is discharged in the form of pulses.

The piston stroke can be set to any required volume; syringes with capacities of, for example, 0·5, 1·0, 2·0 and 10·0 μl can be used for applying the solution. The cannula cannot become blocked with solution because the discharge piston continues its stroke until it comes into contact with the end of the needle. A spring-balanced syringe guide guarantees minimum application force so that no damage occurs to the layer. Also, there is no formation of bubbles inside the syringe when volatile solvents are used. The suction and discharge speeds are uniformly even. After the sample solution has been applied, three wash cycles are automatically switched on by pressing the 'wash' button.

*Further information from Schott Glaswerke, Hattenbergstrasse 10, D 6500 Mainz, FR Germany. Tel.: 0 61 31 66 29 87.*

**Circle No. 14 on Reader Enquiry Card**

### Slimline counter

The latest electronic counter from Trumeter can be installed instantly, regardless of voltage. It is a universal totalizing counter, which is only 16 mm thick and comes ready for surface mounting. It has flying lead connections and is suitable for a supply voltage range from 6V d.c. up to 240V a.c., using a battery or mains supply. Clear, 12 mm high figures read up to 19999; the casing will cover most existing panel cut-outs. The count rate is up to 50 counts/s and the counter has high noise immunity CMOS logic.

*Details from Trumeter Company Ltd, Milltown Street, Radcliffe, Manchester M26 9NX, UK.*

**Circle No. 15 on Reader Enquiry Card**

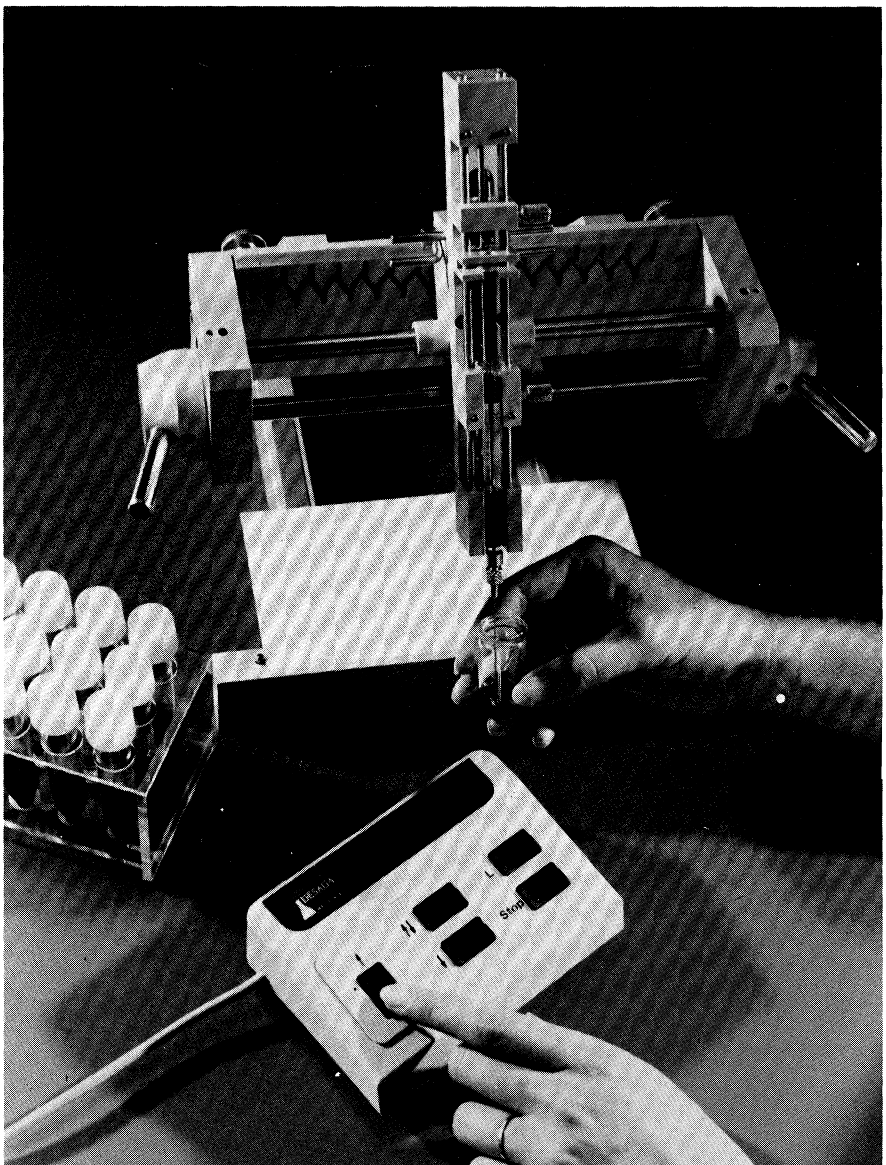

*The PS 01—a semi-automatic applicator for thin layer chromatography from Desaga of Heidelberg.*

### Microprocessor-controlled photometer

Using chemiluminescent and bioluminescence techniques to provide great analytical sensitivity, the Turner Model 20 Photometer is fully automated and gives stable, drift-free operation. Its applications include viable cell assay, activated sludge control, oceanographic research biochemistry, fermentation processes, immunology and research biochemistry. Although a recorder output is available, it is not needed in most conditions: a feature of the Model 20 is an ability to provide, on an LED display, peak, half-integral and full integral information. There is a choice of injector systems utilizing a pipette dispenser for precision and versatility, a multiple dispenser for high volume work or a disposable syringe system to eliminate cross-contamination. A new 200 $\mu$l flow-through cell has just become available, which can be used for both stop-flow and continuous-flow analysis.

*For details of the Model 20 Photometer and its accessories contact Techmation Ltd, 58 Edgware Way, Edgware, Middlesex HA8 8JP, UK. Tel.: 01 958 3111.*

**Circle No. 16 on Reader Enquiry Card**

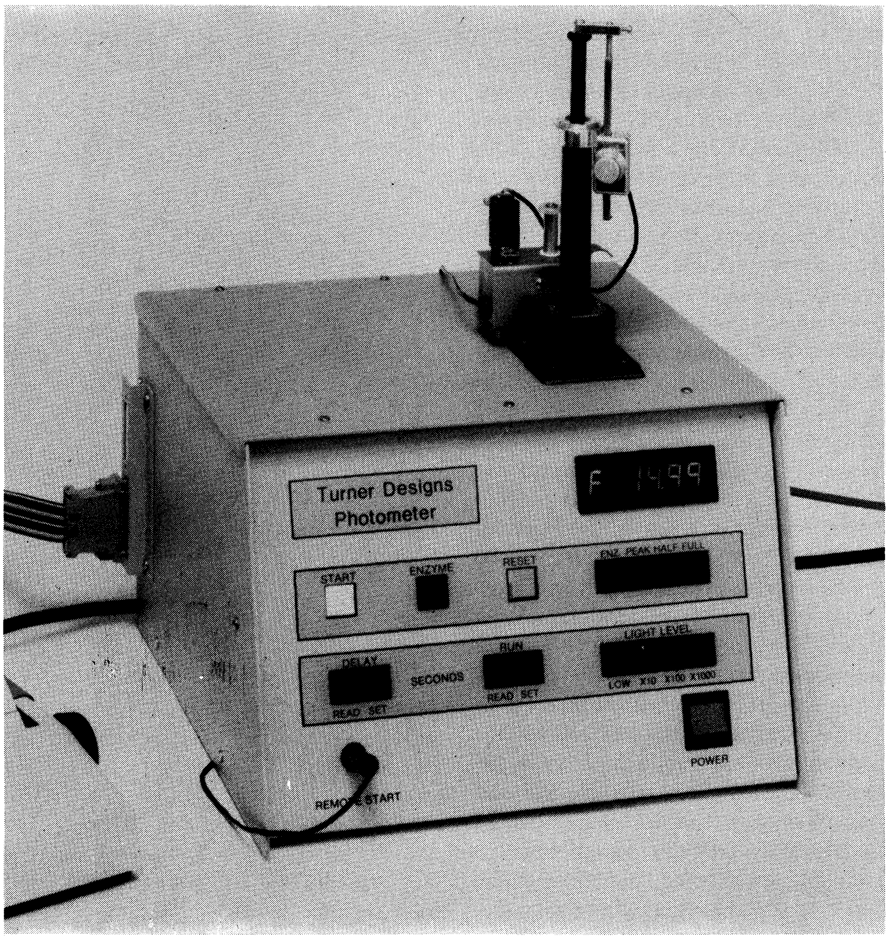

*The Turner Model 20 Photometer; Techmation Ltd, Edgware, UK offer an illustrated brochure describing operation and applications.*

### 'Laboratory Reporter'

Fisher Scientific's 22-year-old house journal offers, in issue 20:3, a survey of some of the methods being used to solve 'tough' environmental problems with such instruments as penetrometers, spectrophotometers, chromatographs, gas proportional counters, and liquid scintillation counters. The issue also highlights some of the latest developments in laboratory products for industrial, government, and educational laboratories.

Among the Fisher instruments featured are: the microprocessor-controlled Model 475 Sulfur Analyzer, which is able to handle a wide variety of sample materials and of eliminating nitrogen oxide and chloride interferences via a patented amperometric titration technique; an all-purpose portable velometer; the new bench-top Isotemp Model 496 Moisture Oven; and a reagent-complete ampoule system—the first ever approved by the EPA—for COD analyses.

*Copies of 'Laboratory Reporter' 20:3 from Fisher Scientific Co., 711 Forbes Avenue, Pittsburgh, Pennsylvania 15219, USA. Tel.: 412 562 8546.*

**Circle No. 17 on Reader Enquiry Card**

### Orion handbook

The 144-page *Handbbook of Electrode Technology* from Orion Research, priced at £5·00, includes a glossary and an appendix on ion-selective electrode technology. The glossary, starting with 'Absolute MV mode' and ending with 'Zirconium in thorium and uranium solutions', defines significant terms, summarizes relevant analytical procedures utilizing ISEs, and provides pertinent bibliographical references, and descriptions of electrodes that are generally available. The appendix section includes overviews on electrode response; such methodologies as standard calibration, titration, incremental methods, and grans' plot; and the variables affecting precise measurements, i.e. reference electrode potential, temperature, ionic strength, sample pH and interferences. Also in the appendix is a cross-reference bibliography organized by electrode, and a table of electrode specifications.

*Orders to Miss Janet Bray, MSE Scientific Instruments, Manor Royal, Crawley, Sussex RH10 2QQ, UK. Tel.: 0293 31100.*

**Circle No. 18 on Reader Enquiry Card**

### HP 3350 explained

Hewlett-Packard's HP 3350 laboratory automation system is featured in two new colour brochures. The first (Publication 5953–1623) describes the computer system capabilities and peripherals available with the series and discusses effective laboratory managment through such system features as expanded memory capability and system security. Two of the company's customers describe their HP 3350 applications and the benefits derived from the system's use.

The second brochure (Publication 5953–1645) provides a technical review of the latest member of the HP 3350 series—the HP 3357. The 19-page booklet features the system's capabilities of data-handling and automation and control of laboratory functions.

*Copies from the Enquiries Section, Hewlett-Packard Ltd, King Street Lane, Winnersh, Wokingham, Berkshire RG11 5AR, UK. Tel.: 0734 784774. (Hewlett-Packard also offer booklets on the HP 5993C and HP 5995B GC/mass spectrometer systems.)*

**Circle No. 19 on Reader Enquiry Card**

### Fail-safe gas detector

The Model 5610 ambient gas detector was launched at the 1983 Petrochemical and Refining Exposition, which was held in Houston at the end of March. The instrument is designed for continuous detection of combustibles and other gases in ambient air. It is explosion-proof, cannot be poisoned by any chemical substance, and has no cross-interference from water vapour.

The 5610 features a unique, active fail-safe system with automatic 'fault' alarm that signals when a malfunction occurs. A function test is automatically initiated every 8 h to check detector sensitivity, calibration and response time. An internal calibration filter is used and no calibration gas is required. It uses the NDIR detection technique with unique non-accumulative zero and span drift, repeatability is better than $\pm 2\%$, and response time is less than 7 s. The single beam system has an automatic compensating circuit for aging and cell window dirt accumulation which ensures low maintenance, stable operation and continuous output. And the open cell design of the 5610 provides a representative ambient sample without a sample system, which means increased cost savings.

*Additional information from Astro Resources Corporation, 100 Park Avenue, League City, Texas 77573, USA. Tel.: 713 332 2484.*

Circle No. 20 on Reader Enquiry Card

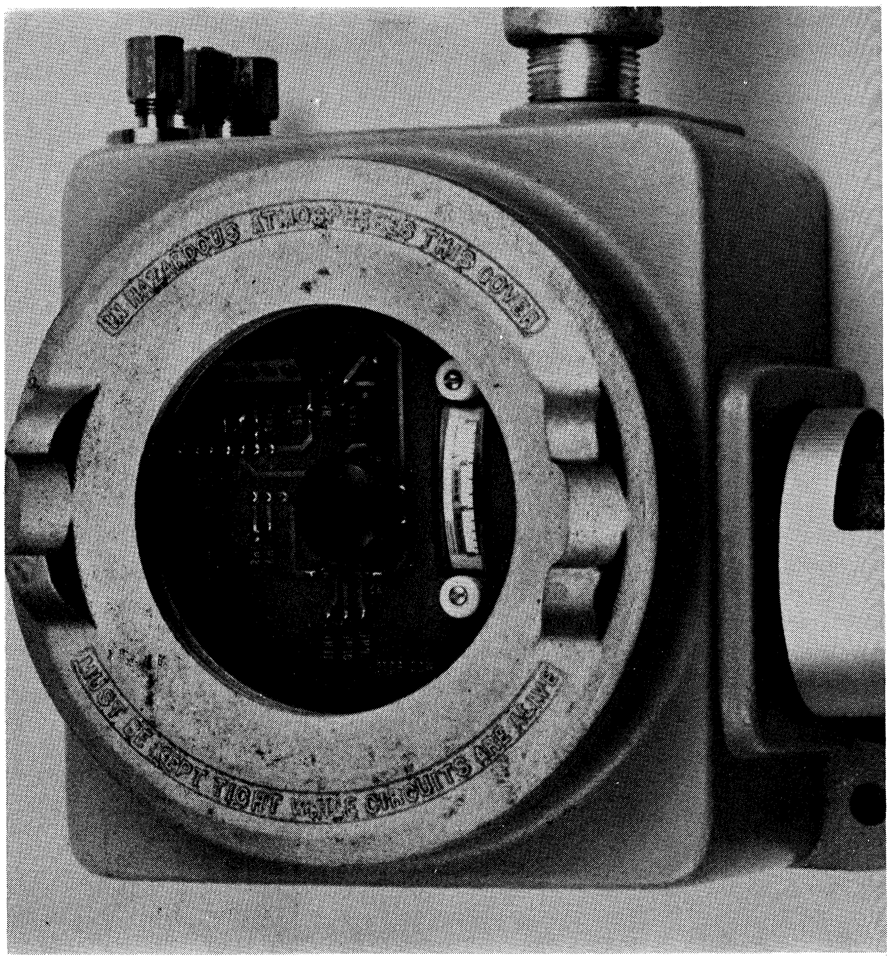

*The Model 5610 ambient gas detector. Safe operation is ensured by a combination of short intervals of active self-testing and an inherent fail-safe mode. (Astro Resources Corporation, League City, USA.)*

### Organic carbon spill detector

Astro Resources have also announced an organic carbon spill detector, the Model 1820. The new instrument offers continuous detection ranges from 0–20 ppm to 0–10 000 ppm as total carbon. Typical samples contain $C_1$–$C_6$ paraffins and olefins, as well as other hydrocarbon complexes. Applications include plant feed-water conditioning, boiler feed, condensate return, heat-exchanger leaks, water run-off, carbon-bed breakthrough, process waste blowdown, and plant discharge. The 1820 incorporates a single-stage sparging column for continuous analysis. The NDIR detection system is sensitized to hydrocarbons and has a $\pm 2\%$ non-accumulative zero and span drift; repeatability is better than $\pm 2\%$, and typical response time is 3 min. Like the company's ambient gas detector, the instrument features an automatic compensating circuit for aging and cell window dirt accumulation to ensure low maintenance, stable operation and continuous output. The alarm package includes a fail-safe alarm, dual level alarms and a 4–20 ma output signal.

*Details from Astro Resources Corporation above.*

Circle No. 21 on Reader Enquiry Card

### Spectrophotometer

Hach Company's DR/3 spectrophotometer combines improvements in the company's DR/2 model with some new features. Electronic range expansion provides greater sensitivity for a variety of tests without the need for expensive accessories, and improved circuitry provides greater reliability and stability over a wider range of environmental conditions.

A portable model, with a carrying case which has room for accessories and reagents, is available with an optional built-in conductivity meter for specific conductance, total dissolved solids and temperature measurements. Operating on batteries or a.c. power, the portable model features a low-battery indicator and can be converted from 115V to 230V a.c.

There is a laboratory model, which includes a 0–1V recorder output permitting a graphic display of results. Slide-in meter scales calibrated in concentration are made from a durable plastic material. Other accessories include a pour-through cell for rapid repetitive sample handling and a 1 cm cell adapter.

Hach can provide reagents and apparatus that allow the DR/3 to measure many water, waste-water and industrial parameters, on-site or in the laboratory.

*For more information write to Hach Company, PO Box 389, Loveland, Colorado 80539, USA. Tel.: 303 669 3050.*

Circle No. 22 on Reader Enquiry Card

